# Welcoming pregnant women: introducing navigation and support services in a secondary health facility in Bangladesh

**DOI:** 10.7189/jogh.15.04097

**Published:** 2025-06-06

**Authors:** Tajrin Tahrin Tonmon, Md Refat Uz Zaman Sajib, Lubna Hossain, Kamrul Hasan, Hassan Rushekh Mahmood, Saraban Ether, A M Rumayan Hasan, Abu Sayeed, A K M Mahmudul Hassan, Haroon Bin Murshid, Sabrina Jabeen, Fariha Azrin, Ema Akter, Fauzia Akhter Huda, Dewan Md. Emdadul Hoque, Shamsuz Zaman, Vibhavendra Singh Raghuvanshi, Shams El Arifeen, Ahmed Ehsanur Rahman, Abu Sayeed Md Hassan, Anisuddin Ahmed

**Affiliations:** 1 International Centre for Diarrhoeal Disease Research, Maternal and Child Health Division, Dhaka, Bangladesh; 2 University of Illinois Urbana-Champaign, Department of Health and Kinesiology, Urbana, Illinois, USA; 3 International Centre for Diarrhoeal Disease Research, Bangladesh, Health System and Population Studies Division, Dhaka, Bangladesh; 4 United Nations Children's Fund, Dhaka, Bangladesh; 5 United Nations Population Fund, Dhaka, Bangladesh; 6 Uppsala University, Global Health and Migration Unit, Department of Women's and Children's Health, Uppsala, Sweden

## Abstract

**Background:**

Maternal mortality remains high in low- and middle-income countries like Bangladesh, driven by preventable causes such as inadequate health care access and delays in receiving care in health facilities, highlighting the need for improved support in health facilities. This study aimed to explore the opportunities and difficulties of introducing ‘Welcome Persons’, a hospital navigation and support cadre for pregnant women, from the perspectives of recipients, patients’ attendance, health care providers, and facility managers.

**Methods:**

This qualitative study was conducted from 15 March to 30 April 2023, in the District Hospital of Gaibandha, Bangladesh. Data were collected through purposive sampling from health care providers, facility managers, ‘Welcome Persons’, pregnant and delivered women, and their attendants, utilising observations, in-depth interviews, focus group discussions, and key informant interviews, and analytical approaches to ensure scientific rigour. Thematic analysis was performed.

**Results:**

The study revealed the positive influence of ‘Welcome Person’ in maternal health care provision by ensuring timely services, preventing outsiders’ interference, as well as contributing to prompt emergency management, specifically for educationally disadvantaged patients. The triangulated perspectives of the participants highlighted the overall acceptance and usefulness of the ‘Welcome Persons’ but also discovered challenges such as a lack of human resources on the night shift, logistical constraints, and potential gender insensitivity by the recipients.

**Conclusions:**

The study findings provided an explicit understanding of the ‘Welcome Persons’ intervention for facilitating maternal health care services and navigation among pregnant women. While they were well-received, addressing identified barriers is crucial for a sustainable positive impact on maternal health care in secondary health facility settings in Bangladesh.

The global burden of maternal mortality ratio (MMR) (223 per 100 000 live births) remains a pressing issue, with the majority (94%) of deaths occurring in low- and middle-income countries (LMICs), particularly in South Asia [1,2]. The disparities in health care service access and quality among LMICs contribute to the high MMR [3,4]. This highlights the need for more patient-friendly maternal care, as emphasised by the global health development agenda, such as the Sustainable Development Goals (SDGs) and Global Strategy for Women’s, Children’s, and Adolescent’s Health, 2016–2030 [5,6].

To reduce the disparities, global health organisations have prioritised addressing the ‘three delays’ of maternal mortality – delay in care-seeking, delay in reaching health care facility, and delay in receiving care at the facility after reaching there [7]. Delays after arriving at facilities are associated with 3–16% of maternal deaths and are strongly advocated for being addressed, particularly in LMICs [8–10]. Other studies also mentioned care delays due to a shortage of trained care providers, poor management and coordination of care provision, long waiting times, poor navigation within facilities and outsiders' manipulations in decision-making as crucial causes of maternal death and adverse outcomes [10,11]. Notably, 80% of maternal and perinatal deaths are preventable with the timely provision of maternal health care when sought [12].

Like other LMICs, Bangladesh has a high MMR (196 per 100 000 live births), irrespective of a noteworthy decline in recent times [4,12,13]. Here, the lack of timely and quality care for pregnant women in health facilities continues to be a challenge. Also, service provision, capacity, and quality of care in public facilities remain sub-optimal [14,15]. In such a context, patient facilitation and navigation support can improve maternal health care by addressing inequalities in care-seeking, minimising delays, and improving patients’ experiences. However, such initiatives are less common in women's health care, especially in LMICs, including Bangladesh [16,17]. Thus, a distinct support cadre, ‘Welcome Persons’, was introduced in the Gaibandha District Hospital between April 2022–June 2023. The ‘Welcome Persons’ were local people who were studying or completed Bachelor’s degrees as well as received intensive training on emergency maternal health care from the study physician before deployment. Their role included but was not limited to supporting and navigating pregnant women throughout their journey in every step of the hospital, starting from the emergency department to all other places where pregnant women may need to seek services (*e.g.* antenatal care corner, labour ward, surgery room, if needed), notify duty doctors and nurses, facilitate laboratory procedures and referrals and even escort pregnant and delivered women to the vehicles upon discharge, ensuring a smoother hospital experience. They usually worked in three shifts by rotation with monitoring of the ward masters: morning (8 am–2 pm, four pax), evening (2 pm–8 pm, two pax), and night (8 pm–8 am, two pax).

Despite incorporating similar support cadres in different health care domains previously in Bangladesh and beyond, very few studies have attempted to explore the health system integration aspect of similar support cadres [18–22]. Moreover, most of these studies focused on patient preferences for the type of support and support person. However, no study has specifically assessed the viability and practical challenges of such intervention, especially from relevant beneficiaries and stakeholders’ perspectives such as pregnant women, health care providers, and facility managers. Therefore, this study aims to explore the opportunities and difficulties of introducing ‘Welcome Persons’ from the perspectives of recipients, patients’ attendance, health care providers, and facility managers. Additionally, it explores their recommendations for integrating a supporting cadre into the current health system of Bangladesh.

## METHODS

### Study design and site

This qualitative study employed an ethnographic design, combining participant observation and interviews at the Gaibandha District Hospital. Ethnography is well-suited for the study to explore the complexities of facility practices with the intervention for such a key demographic group [23]. Key Informant Interviews (KIIs) were conducted with facility managers and health care providers, and In-depth Interviews (IDIs) with pregnant women, delivered mothers, their attendants, and the in-charges of the maternal ward. Focused Group Discussions (FGDs) were conducted with nurses and ‘Welcome Persons’. All the participant observations, interviews, and FGDs were conducted between 15 March–30 April 2023.

Gaibandha District Hospital is a secondary-level health care facility in Gaibandha district of Rangpur Division in Bangladesh. It was reportedly one of the under-performing district hospitals among the eight districts of the Better Health District Model project in terms of providing maternal health care services [24,25]. The selection of the Gaibandha District Hospital for introducing ‘Welcome Persons’ was in accordance with the suggestions of the managers of the Maternal Health programme of the Government of Bangladesh and national stakeholders who are experts in the Maternal and Newborn Health domain.

### Study participants and sample selection

With permission and active help from the hospital authority, we used a purposive sampling technique for selecting health care providers (n = 8), facility managers (n = 4), ‘Welcome Persons’ (n = 8), pregnant and delivered women (n = 10), and their attendants (n = 5). Inclusion criteria for pregnant women for IDIs (n = 5) included pregnancy history (prioritising multiparous patients), previous maternal health care-seeking history from the hospital, and admission duration (at least two days). Facility managers (n = 4) and two in-charge (antenatal care room and labour room) were selected for KIIs and IDIs, respectively, based on their involvement in monitoring and decision-making of the care delivery for pregnant mothers. Two FGDs were conducted with senior staff nurses (SSN) of maternity wards and ‘Welcome Persons’.

### Data collection and interview contents

Participant observations of the recipients, their attendants, health care providers, facility managers and ‘Welcome Persons’ were conducted during hospital office hours to understand the clinical practice and patient-provider interactions. For pregnant women, nurses helped in recruiting. After providing study details and obtaining oral consent, their interactions with the health care providers were observed (n = 25). Participant observation (total of 13 days) also formed the basis for establishing rapport with the hospital employees and creating trust in the researchers, who were ‘outsiders’ to the hospital setting. After each observation, detailed field notes were written, covering informal interviews, observed consultations, the physical environment, and so on. All the IDIs, KIIs, and FGDs were conducted separately at the participants’ convenience.

Interviews followed theme-based guidelines with open-ended questions. All interview tools were finalised after pretesting (six pilot KIIs and IDIs and one pilot FGD), and these data were utilised to create prospective codes for thematic analysis. The IDIs, KIIs and FGDs covered various aspects, including demographics, pregnancy and care history, attitudes and perspectives towards ‘Welcome Persons’, perceived changes in support seeking within the facility and service attainment, including ANC and Postnatal Care (PNC) services from the lenses of barriers and enablers.

The sample size followed saturation principles, with additional interviews until no new information emerged, as ensured by debriefing sessions. Data were collected by experienced qualitative researchers trained in anthropology who were involved in guideline and protocol development, ensuring consistency with the study's objectives. Interviews were conducted in the local language in private rooms, maintaining participants’ privacy and research ethics. For FGDs, the chairs were arranged in a roundtable to promote eye contact. The moderator, assisted by a field research officer, organised sessions, coordinated participants’ schedules and took notes. The moderator sat facing the door while another investigator played the gatekeeper role.

### Data management and analysis

All IDIs, KIIs, and FGDs were audio-recorded and transcribed Verbatim in Bengali. Then, the transcription was translated into English, and thematic analysis was carried out [26]. These analytical procedures were undertaken in six stages:

a) becoming familiar with the transcribed data through repeated readings

b) generating initial codes and gathering data under each code

c) identifying themes and sub-themes and extracting data into themes and sub-themes related to environmental and social barriers to healthy food

d) reviewing themes and formatting a thematic matrix for further analysis

e) defining and naming themes

f) write up [27].

A preliminary codebook was developed based on a thorough review of transcripts and observational field notes. These codes were finalised upon team consensus. Three researchers separately coded all the transcripts using ATLAS.ti, version 7.5.7 (ATLAS.ti Scientific Software Development GmbH, Berlin, Germany). Team members discussed their understanding and disputed inconsistencies at every step, including any coding disagreements in the presence of other researchers, to ensure the validity and consistency of the coding [28].

Data collection and analysis were done simultaneously. While analysing, researchers employed a systematic approach involving periodic meetings to review and discuss coded data to ensure inter-coder reliability. These sessions served to address ambiguities and update the codebook as needed, ensuring clarity and consistency in the coding framework. Coders actively engaged in discussions to interpret the data and justify the specific codes. These deliberations helped identify whether disagreements arose from ambiguities in the data, inconsistencies in the coding framework, or differences in interpretation. Instances of agreement and disagreement were thoroughly examined and resolved collaboratively, strengthening the reliability of the findings [29,30]. All transcripts, codes, subthemes and theme categories were rechecked, and data labelling was done. Through triangulation of multiple data sources (data from different types of stakeholders), investigators (separate researchers for different groups), collection methods (participant observation, KII, IDI and FGDs), and analytical approaches were attempted to ensure scientific rigour. This involved revisiting raw data, interview transcripts, field notes, and observation notes to ensure accurate interpretation and identify subtle nuances that might reconcile the conflicts. Additionally, peer debriefing sessions were held to share findings and perspectives.

### Outcome area

**Figure 1:**
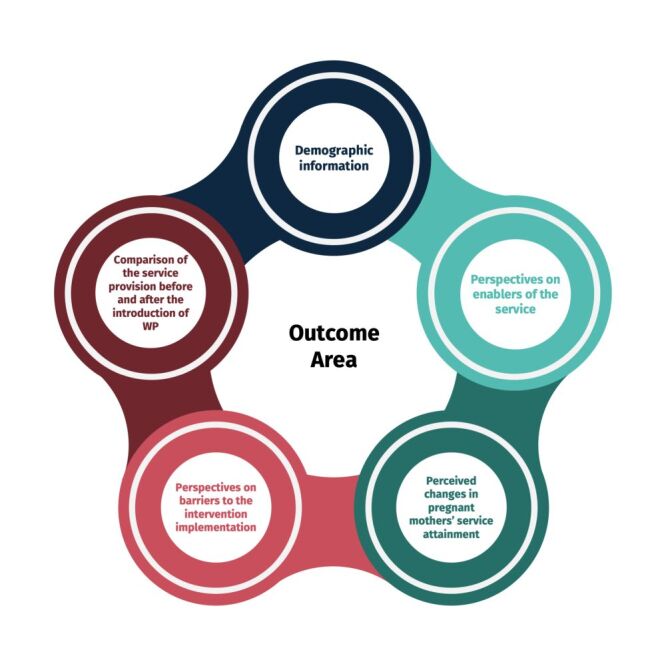
Outcome areas of the study.

The study focused on different outcome areas based on the characteristics and interactions with participants, as well as study objectives (**Figure 1**).

## RESULTS

The overall findings revealed participants’ characteristics, enablers and barriers to the intervention, as well as the recommendations/suggestions for further integration, with several sub-themes and exploration areas (**Table 1**).

**Table 1:** Summary of findings: exploration area and key codes

Exploration areas	Emerging key codes
Demographic characteristics	Age
	Sex
	Education
	Occupation
Enablers of the service of the ‘Welcome Persons’	Reduced waiting times
	Efficient patient navigation
	Ease in service provision due to the active role of ‘Welcome Persons’
	Emergency management
	Countering manipulative outsiders’ trickery
	Behavioural decency
	To make understand educationally disadvantaged people
	Access to clinical laboratory for investigation
	Significance of Welcome Persons’ assistance
Barriers to the service of the ‘Welcome Persons’	Gender sensitivity
	Limitation of manpower
	Additional workload beyond their job responsibilities
Recommendations	Integration in the current secondary health facility organogram

### Demographic characteristics of the participants

Demographic characteristics varied across different categories (**Table 2**). The study included a total of 35 respondents for KIIs, IDIs, and FGDs. Among the respondents, around 65% were female and 35% were male. The majority of the respondents (51.4%) were within the 26–35 years age group. Regarding educational qualification, around 65% had a bachelor's degree or above, while 17% had secondary education. Additionally, 57.4% of the respondents were employed in the service sector, and 28.6% were housewives.

**Table 2:** Participants’ characteristics by the interviewing types

Characteristics	Pregnant women	Delivered women	Attendants	Facility managers	Health care Providers	‘Welcome Person’	Total, % (n = 35)
Sex							
*Male*	0	-	3	3	0	6	12 (34.3)
*Female*	5	5	2	1	8	2	23 (65.7)
Age (years)							
*18–25*	3	2	-	0	-	2	7 (20)
*26–35*	2	3	3	2	3	5	18 (51.4)
*36–45*	0	0		0	2	1	3 (8.6)
*46–55*	0	0	2	2	3	0	7 (20)
Education							
*Primary (grade 1–5)*	0	2	1	0`	0	0	3 (8.8)
*Secondary (grade 6–10)*	3	2	1	0	0	0	6 (17.1)
*Higher Secondary (grade 11–12)*	1	-	2	0	0	0	3 (8.6)
*Bachelor or above*	1	1	1	4	8	8	23 (65.7)
Occupation							
*Service*	0	0	-	4	8	8	20 (57.4)
*Business*	0	0	3	0	0	0	3 (8.8)
*Housewife*	5	3	2	0	0	0	10 (28.6)
*Others*	0	2	-	0	0	0	2 (5.7)

### Enablers of the service of the ‘Welcome Persons’

According to the findings, ‘Welcome Persons’ were generally accepted by pregnant women, their attendance, health care providers and facility managers. Helping intentions, welcoming gestures, faster patient admissions and adept delay management were widely appreciated by all the participants. The gynaecologist also relied on them for patient assistance and management, fostering trust and making them the first point of contact for patients facing difficulties.

‘Welcome Persons’ significantly reduced waiting times for maternal health care and facilitated efficient patient navigation from arrival to discharge. Facility managers mentioned a noticeable improvement since ‘Welcome Persons’ implementation, echoing perspectives from most of the care recipients, who appreciated the help received at the entrance. According to one of the facility managers:

*Of course, they (‘Welcome Persons’) have brought changes, particularly in time efficiency. Previously, pregnant mothers would come into the emergency department and be confused about the route to the labour ward, which would cost them almost half an hour to reach the desired ward. She might even mistakenly go to another ward and return after roaming here and there, wasting much time. But now it is saved as ‘Welcome Persons’ receive and accompany them till the labour ward, and so the treatment begins faster than before.* – 35 years, facility manager, physician

One of the service recipients highlighted how prompt navigation and timely service facilitation eased their stay in the hospital, among other appreciations:

*This is my first time in this hospital. I did not know where to go or whom to talk to. When I told him (‘Welcome Person’) that I arrived with labour pain for delivery, he instantly managed to buy the ticket. Afterwards, I saw him completing my paperwork, explaining my purpose to the emergency doctor, and he was there with me until my bed was set for lying down… He was really helpful.* – 19 years, pregnant woman, secondary education, housewife

Moreover, the health care providers expressed ease in service provision due to the active role of ‘Welcome Persons’ in fetching patients’ delivery reports and bringing them timely. One of the FGD participants indicated this, and others agreed:

*And ‘Welcome Persons’ also assist in collecting patients’ test reports quickly; patients can't do it faster by themselves, so they (‘Welcome Persons’) bring it faster. Every minute counts when a pregnant woman is in severe labour pain and ‘Welcome Persons’ ensuring the health care providers receive patients’ test reports timely hastens the treatment.* – 53 years, health care provider, diploma in nursing

In case of emergency management, ‘Welcome Persons’ assistance was specifically appreciated and found effective. They helped critical patients with postpartum haemorrhage (PPH), severe anaemia, pre-delivery blood transfusion, and labour pain or abdominal pain. They provided blood bank-related information and navigated patients towards the care-seeking points, especially those who were unfamiliar with the hospital setup. The nurses of the labour room mentioned:

*Earlier, I even saw patients coming with severe pain with agitation, and aggression; finally arriving at the labour ward after wandering around in the hospital for around 20–30 minutes. Cases were found, in fact, being delivered on the second floor in the corridor of the general ward after spending time to find the labour ward. And now, patients reach the labour ward within 5–10 minutes, and we do not find them complaining about any hustle. Luckily, ‘Welcome Persons’ are there to navigate or accompany.* – 40 years, health care provider, MSc in nursing, 2nd year, diploma in midwifery

Another frequently mentioned enabling factor was ‘Welcome Persons’ active role in safeguarding patients from outsiders’ or brokers manipulation. Two-thirds of all the participants specifically acknowledged the influence of the outsiders in this hospital and ‘Welcome Persons’ roles to change the scenario. One of the facility managers said:

*This, I am talking about the labour ward; they safeguard the pregnant women here. Now let me tell you what happens to other wards; too many outsiders barge into those wards and manipulate innocent patients. But having ‘Welcome Persons’ here, no manipulative outsider can enter anymore; I am very satisfied, and I mean it. Even if the outsiders try to influence them, ‘Welcome Persons’ stop them immediately. It’s a relief.* – 48 years, facility manager, physician, postgraduate surgical specialist

Irrespective of first-time or multiple times hospital experiences, most care recipients responded aligned with the above findings. The following quote from a care recipient illustrates this underlying view:

*They are providing good services, indeed. Because my personal wish was to have a normal delivery; my gynae also told me that I would have a normal delivery. But an outsider kept saying that having a normal delivery here would be a huge life risk for my baby and that I should have a C-section delivery from any outside clinic as the service here is not good enough. However, the ‘Welcome Persons’ convinced me by saying that normal deliveries are happening here very frequently and not to worry about the quality of services. Still, if otherwise, the decision should be taken by the gynae after examining me... …Now, you can see me with my baby beside me; I’m recovering here after having a normal delivery.* – 27 years, pregnant women, master’s degree, housewife

Among other enablers, ‘Welcome Persons’ behavioural decency was one of the highly appreciated findings as mentioned as a reason for their wide acceptance.

One pregnant woman mentioned:

*It felt nice; they are well-behaved. If they weren’t good people, they wouldn’t help me all these times, would they? No aggression or resentment was there; in fact, it felt, as if they were one of us, so simple and easy to reach… that brother (‘Welcome Person’) even fetched my family members from the entrance when they wanted to visit me. My sister said that she rarely receives such cheerful service…* – 20 years, pregnant woman, secondary education, housewife

For educationally disadvantaged people, understanding the care-seeking process of the hospital poses additional challenges, leading to being neglected in receiving necessary services [31]. ‘Welcome Persons’ have ensured that regardless of patients’ or attendants’ literacy level, they can get proper maternal health care according to the facility protocol and do not return without receiving it. One of the service recipients with limited formal education mentioned great dependency and trust in ‘Welcome Person’:

*I know I can come to them and ask anything if I face any problem; at least they will show me a way like they escorted me right from the road and brought me here (labour ward). If for a less educated person like me, this is an advantage, then it’s an even better service for uneducated ones.* – 20 years, pregnant woman, secondary education, housewife

All the facility managers, health care providers and some care recipients indicated the significance of ‘Welcome Persons’ assistance in progressing the necessary maternal health care services such as ANC and PNC. Specifically, the health care providers and facility managers highlighted their counselling efficacy in bringing satisfactory growth in the number of ANC and/or PNC visits and laboratory tests. The Superintendent of the hospital mentioned:

*Of course, there are changes, thanks to ‘Welcome Persons’ counselling, what used to be 50% has now become 90%...Counselling is the key. They work in ANC corner and help convince the mothers, along with our SSNs’ guidance, to do tests in our laboratory.* – 50 years, facility manager, physician

Around two-thirds of the participants mentioned that before being informed by the ‘Welcome Persons’, they were almost unaware of the existence of a clinical laboratory in the hospital. One of the service recipients mentioned:

*For example, I needed to go for a PNC checkup after discharge. So, they took me from the labour ward after delivery for PNC, which I felt was a help…I mean, I studied (diploma in midwifery), so I was already aware of the services in the hospital. Might I be uneducated, I wouldn't have any clue about PNC unless they (‘Welcome Persons’) would inform me. So, I think, by them, unaware mothers can be benefitted indeed.* – 27 years, delivered mother, diploma in midwifery, ex-midwife

### Barriers to the service of the ‘Welcome Persons’

Despite the enabling factors, several challenges to ‘Welcome Persons’ intervention emerged from the observations, interviews, and discussions. Many of these challenges ‘Welcome Persons’ faced during the initial stage were later resolved with the help of hospital administration.

One such issue, gender sensitivity, was emerged indirectly from the findings. Considering the risks of gender-based violence, female ‘Welcome Persons’ were not assigned during night shifts. This induced some minor challenges as gender plays a vital role in terms of comfort when it comes to pregnant women. One of the patient’s attendants mentioned:

*My daughter (patient) got their services almost all day long. However, at night, I found no sisters (indicated female ‘Welcome Persons’) around. I mean, they are all constantly there in the daytime, but hardly any female staff are seen at night!* – 55 years, patient attendant, primary Education, housewife

Furthermore, the time span from the entrance to the labour ward and the patients’ further stay period was sheer shorter, which imposed challenges for male ‘Welcome Persons’ in creating rapport with patients to overcome the murmured gender insensitivity.

Also, as half the number of ‘Welcome Persons’ were assigned during night shifts compared to the daytime, a temporary void had been created in the emergency department when the allotted ‘Welcome Persons’ would navigate pregnant women to the service points. However, many of them worked nearly 12-hour shifts due to excessive patient flow. Thus, most participants requested to increase the number of people working, especially at night shifts. This has also been highlighted in the ‘Welcome Persons’ FGD: *As we have less manpower in night shifts, it gets stressful sometimes. At midnight, around 3 or 2 am, patients keep coming and coming in. The nurses are usually at rest around 3 am”* – 22–40 years, Bachelor’s Degree Studying or completed, ‘Welcome Persons’

Considering the limited resource setting in a district hospital, the demand for services is often beyond the capacities and job responsibilities of the health care providers. ‘Welcome Persons’ were no different from that. Additionally, ‘Welcome Persons’ helping nature sometimes induced additional engagements beyond their job responsibilities, costing a great deal of time and excessive physical labour. Moreover, the lack of logistical support further exacerbates the burden.

Regarding this, the ‘Welcome Persons’ shared in the focus group discussion:

*Often, patients with critical conditions come. Once, a patient with PPH came at night, so I ran immediately to manage blood, but could not find a matching blood group anywhere in the nearby facilities, let alone our hospital. Being concerned, I reached out to all ‘Welcome Persons’ and circulated this information on every social platform, including the blood bank groups we are associated with in our own lives. Finally, I found a donor from my college’s blood bank group and proceeded with the needful… In such cases, we gather the patients’ information immediately, inform the in-charge and start doing the needful. –* 22–40 years, Bachelor’s Degree Studying or completed, ‘Welcome Persons’

One of the pregnant women mentioned:

*I could not find a matching blood group. So, one of them (‘Welcome Persons’) went to my village to find a matching donor and even managed to drop him off at his home after he donated. They are really good people, and my heartiest blessings to them.* – 35 years, pregnant woman, primary education, tailor

Sometimes, ‘Welcome Persons’ (with degrees from Medical Assistant Training Schools) had to help health care providers by checking blood pressure and arranging oxygen for the patients to manage the huge patient flow. One of the SSN mentioned:

*They help me in ANC corner. The girl here in duty has a MATS (Medical Assistant Training Schools) degree and checks diabetes and blood pressure of pregnant women in rushed hours when there are a lot of patients in the queue.* – 56 years, health care provider, diploma in Nursing and midwifery

Regarding such additional induced responsibilities, one of the ‘Welcome Persons’ mentioned logistical constraints as one of the underlying reasons:

*We mostly carry patients to the labour ward on the trollies, pushing it all by ourselves. Sometimes, their labour pain seems to be so profuse as if they would die immediately. One day, a patient with excessive bleeding came, but we could not find a trolley as there was only one trolley in the hospital. So, we managed a van (a three-wheeled human-pulled vehicle used for transport) to carry her, pushed the van to the labour ward and put a slack under her to stop the blood from spreading everywhere. … Lucky that patient, survived and delivered.* – 24 years, Bachelor’s, 3rd year, ‘Welcome Person’

### Recommendations

Given the noticeable engagement of ‘Welcome Persons’ with pregnant women and delivered mothers, as well as favourable changes in service facilitation and navigation, health care providers and facility managers mentioned this intervention as timely and persuasive. In a situation of excessive rush and high service demand for maternal health care in the secondary hospital setting, participants emphasised that such an additional support cadre could be extremely valuable and should be introduced in other similar settings. The Superintendent of the facility mentioned:

*With such a limited workforce, it is almost impossible to manage such a huge workload. They are playing a significant role in providing quality services in the hospital, and I can say that very strongly… So, I wish these ‘Welcome Persons’ longer stay in the hospital. They are changing the momentum in the care provision of pregnant women, and my care providers are very satisfied with them, too. That is why I want such skilled manpower to be established not only for my hospital but also in other hospitals in the country.* – 50 years, facility manager, physician

However, they pointed out the number of ‘Welcome Persons’ in action seemed insufficient, which could lead to service disruptions during peak hours, potentially resulting in patient dissatisfaction and disparities in service. The labour word in charge mentioned:

*In my ward, I see they (‘Welcome Persons’) are always active and stand by to serve the pregnant mothers. But, when one care provider is engaged somewhere else, another needs to fill in the void. Otherwise, it is not feasible to attend to everyone and run everywhere. Hence, I believe it’s surely a great initiative for this hospital and will benefit other hospitals too.* – 40 years, health care provider, MSc in nursing, 2nd year, diploma in midwifery

## DISCUSSION

This qualitative study explored different aspects of introducing the distinct dedicated service cadre ‘Welcome Persons’ in one of the district hospitals of Bangladesh to facilitate maternal health care services from the triangulated perspective of facility managers, health care providers, and care recipients. Interviews with these multi-level stakeholders revealed that the ‘Welcome Persons’ positively influenced care-seeking by ensuring timely health care services, refrained outsiders’ conspiracy, and providing robust assistance in patients’ emergencies, *e.g*. blood management, faster transportation of pregnant women, with special focuses on serving educationally disadvantaged patients as well as increasing laboratory, ANC and PNC services. Moreover, their simple, decent behaviour and helping attitude were widely appreciated. However, among several challenges, the lack of female ‘Welcome Persons.’ specifically at night shifts, overall human resource shortage at the hospital, induced additional workloads, and logistical constraints causing excessive physical labour were highlighted.

The positive influence and extent of ‘Welcome Persons’ were multifold. First, participants uniformly supported the timely health care service as ensured by the ‘Welcome Persons’, aiding in navigation, helping with test reports, accompanying patients to the labour ward, and so on, effectively reducing delays at different service points. Studies in India and Malawi highlighted the importance of time-saving in service points to reduce maternal death [32–34]. In our study, we found that ‘Welcome Persons’ are actively contributing to reducing the delay in receiving care after patients reach the facility, emphasising the necessity of such a supporting cadre in secondary health care settings. This finding aligns with a previous similar intervention for neonatal and early childhood syndromic sepsis management at the sub-district level in Bangladesh [22]. Second, ‘Welcome Persons’ played a crucial role in protecting pregnant women from outsiders’ manipulation regarding service-seeking and decision-making, hence enhancing patients' safety – a critical aspect of health care service in similar settings, given previous evidence of unethical practices [35]. Third, their decent and accepting behaviour, contrasting with many reports of mistreatment by other hospital staff in similar settings, fostered trust among the stakeholders, reinstalling a foundation of faith between the health care providers and recipients [35]. Fourth, ‘Welcome Persons’ contributed to managing emergency situations by facilitating improved utilisation of blood bank and laboratory services, which are crucial for addressing delivery complications such as PPH [13,34,36,37]. Moreover, in such situations, ‘Welcome Persons’ active participation helped the health care providers to better manage patient flow and crowd, facilitating quicker diagnosis and management. Fifth, ‘Welcome Persons’ counselling has shown a proven increase in ANC and PNC visits, addressing barriers such as patients’ reluctance, as reported by other studies [38]. Finally, their services across care points ensured comprehensive support for the care-seeking pregnant women, specifically benefitting educationally disadvantaged patients and their attendants, addressing one of the key challenges in health care-seeking in Bangladesh [39]. Overall, ‘Welcome Persons’ significantly enhanced stakeholders’ experiences, highlighting the sustainable impact of their presence.

Regardless of the enablers, the ‘Welcome Person’ intervention faced significant challenges. The distinct establishment of a ‘Welcome Person’ as a support cadre in the district hospitals may be hindered by gender-related hesitancy and cultural norms, according to our participant's observations as well as previous studies [40]. Thus, targeted recruitment strategies should involve community consultations and consider local gender dynamics for increased acceptance and impact [41]. Emphasising gender-sensitivity modules during the ‘Welcome Person’ training, additional gender-sensitisation training for the existing hospital staff and community, and subsequently assigning female ‘Welcome Persons’ during night shifts (necessarily in pairs/more than one per shift) could foster inclusivity, reduce gender hesitancy, and increase necessary support availability and comfort for pregnant women. Additionally, logistical shortages, a common scenario in limited resource settings like Bangladesh, resulted in excessive physical labour for the ‘Welcome Persons’, specifically the limited number of trollies for patient transportation, exacerbating their regular workload. Facility managers and responsible government entities should prioritise funding for patient transportation tools such as trolleys as well as establish a dedicated maintenance fund to ensure their around-the-clock availability. Moreover, as evident in other studies, an inadequate number of staff during night shifts (both hospital staff and ‘Welcome Persons’) causes excessive workload and frequent temporary voids in the emergency departments, underscoring broader health care resource concerns such as a low physician-to-patient ratio (0.6 physicians per 1000 patients) in Bangladesh [32,38,42–44]. One possible solution could be to increase shift-based part-time workers, which could be facilitated within the current contract worker model in the facilities. Furthermore, based on the experience of involving in additional tasks beyond their defined responsibilities, the work responsibilities of the ‘Welcome Persons’ should be redefined and structured during future implementations, as well as consider the fact of a low resource setting with high service demand in a secondary hospital.

In general, the participants strongly advocated for the ‘Welcome Persons’, with facility managers and health care providers expressing unanimous support for their introduction nationwide to enhance maternal health care among health facilities. Service recipients overwhelmingly acknowledged the idea of introducing such a supporting cadre in all hospitals, particularly for pregnant women and newborns, highlighting the need and feasibility of such intervention. Moreover, the already established Quality Improvement Secretariat under the Ministry of Health, Government of Bangladesh, focusing on maternal and newborn health, can easily incorporate such a supportive cadre, considering its non-technical approach to streamline patient flow and reduce delays. However, the challenges of high patient load with insufficient human resources were overly acknowledged in the KIIs.

This paper should be interpreted in the light of its strengths. First, we incorporated a comprehensive sample including relevant stakeholders (facility managers, health care providers, care recipients), as well as, utilised multiple data collection methods, including interviews, discussions and observations. The ratio of both male and female participants was almost equal, ensuring gender representation, and the Gaibandha district hospital represents a secondary resource constraint hospital setting in Bangladesh. Moreover, the strongest feature was the triangulated result construction in terms of methods, data sources and investigators. Despite these strengths, the study demonstrated a few limitations, such as the potential for design or selection bias due to implementing the new cadre in only one district hospital. Although this contributes to the lack of scope for mass generalisability, this qualitative study was not intended to create generalisability but rather to explore the opportunities and challenges of introducing and larger piloting such support cadre in Bangladeshi and similar settings. Also, other confounding variables, such as changes in hospital management as usual procedures of government human resource management and the influence of external health care organisations and policies, were not considered. Moreover, the Hawthorne effect could be another limitation where participants alter their behaviour due to awareness of being observed or studied. While efforts were made to mitigate its impact – such as prolonged engagement with participants, fostering a neutral and comfortable research environment, and employing reflexivity during data analysis – participants' awareness of being observed may still have influenced their behaviour; however, it is unlikely to persist after the ample implementation time [45]. Finally, the fear of job uncertainty of the ‘Welcome Persons’ and the biased perspectives of the care providers due to established rapport may cause potential information bias.

## CONCLUSIONS

The ‘Welcome Persons’ received an overall positive response from hospital management to health care providers and care recipients in facilitating maternal health care management in a Bangladeshi district hospital. Care providers acknowledged the usefulness of the ‘Welcome Persons’ in supporting emergencies about clinical management and as a comprehensive way to provide structured and informal assistance in providing maternal health care to ease and fasten the service attainment of hospital care-seeking pregnant women. Moreover, the care recipients and their attendants encouraged the implementation of ‘Welcome Persons’ delightfully. Following addressing the mentioned barriers, such a supporting cadre can possess a potential positive influence in the case of maternal health care in Bangladesh and other similar settings.
